# Association of tumor and plasma microRNA expression with tumor monosomy-3 in patients with uveal melanoma

**DOI:** 10.1186/s13148-016-0243-0

**Published:** 2016-07-22

**Authors:** Pierre L. Triozzi, Susan Achberger, Wayne Aldrich, John W. Crabb, Yogen Saunthararajah, Arun D. Singh

**Affiliations:** Taussig Cancer Institute, Cleveland Clinic Foundation, Cleveland, OH USA; Cole Eye Institute, Cleveland Clinic Foundation, Cleveland, OH USA; Wake Forest School of Medicine, Medical Center Boulevard, Winston-Salem, NC 27157 USA

**Keywords:** Prognosis, Biomarkers, miR-92b, miR-199-5p, miR-223

## Abstract

**Background:**

Epigenetic events mediated by methylation and histone modifications have been associated with the development of metastasis in patients with uveal melanoma. The role of epigenetic events mediated by microRNA (miR) is less clear. Tumor and plasma miR expression was examined in patients with primary uveal melanoma with tumor monosomy-3, a predictor of metastasis.

**Results:**

miR profiling of tumors by microarray found six miRs over-expressed and 19 under-expressed in 33 tumors with monosomy-3 compared to 22 without. None of the miRs differentially expressed in tumors with and without monosomy-3 was differentially expressed in tumors with and without tumor infiltrating lymphocytes. Tumors manifesting monosomy-3 were also characterized by higher levels of *TARBP2* and *DDX17* and by lower levels of *XPO5* and *HIWI*, miR biogenesis factors. miR profiling of plasma by a quantitative nuclease protection assay found elevated levels of 11 miRs and reduction in four in patients with tumor monosomy-3. Only three miRs differentially expressed in the tumor arrays were detectable in plasma. miRs implicated in uveal melanoma development were not differentially expressed. Elevated plasma levels in patients with tumor monosomy-3 of miR-92b, identified in the tumor array, and of miR-199-5p and miR-223, identified in the plasma array, were confirmed by quantitative real-time polymerase chain reaction. Levels were also higher in patients compared to normal controls.

**Conclusions:**

These results support a role for epigenetic mechanisms in the development of metastasis in patients with uveal melanoma and the analysis of miRs as biomarkers of metastatic risk. They also suggest that potentially useful blood miRs may be derived from the host response as well as the tumor.

## Background

Uveal melanoma is a rare cancer that leads to metastatic death in up to half of patients. That loss of chromosome 3 in tumors is associated with the development of metastasis is well established, and a variety of techniques are being used to test tumors for monosomy-3 [1]. Gene expression profiling (GEP) has also been effectively applied to characterize tumors with a high risk of metastasis, “class 2,” and tumors with a low risk, “class 1” [2]. Epigenetic events have also been implicated in uveal melanoma metastasis. When independently analyzed for global DNA methylation profiles, primary uveal melanomas cluster into two groups that are identical to the class-2 and class-1 groups identified by GEP [3]. Expression levels of a number of histone-modifying genes and polycomb family members are significantly lower in uveal melanoma with monosomy-3/class-2 GEP [4]. Although epigenetic events mediated by microRNA (miR) have been implicated in uveal melanoma development [5–7], a role for miRs in the metastatic process has not been established. Worley et al. found six miRs to be upregulated in 12 tumors expressing high-risk, class-2 GEP and 68 to be upregulated in 12 tumors expressing low-risk, class-1 GEP [8]. The most significant discriminators of class 2 were upregulation of let-7b and miR-199a. In contrast, Larsen et al. found no association between miR-expression profiles and histopathological features, staging, metastasis, or survival in a study of 20 patients [9].

Obtaining uveal melanoma tumors for genotyping can be problematic. There is a need for blood biomarkers [10]. miRs are very stable in blood due in part to their incorporation into microparticles and exosomes, and serum and plasma levels of miRs are also under investigation as diagnostic and prognostic biomarkers in cancer and other diseases [11]. Blood miR levels have not been previously reported in uveal melanoma. We used tumor monosomy-3 as well as tumor and plasma miR profiling as guides as to develop miR-based prognostic blood biomarkers for patients with primary uveal melanoma. We also examined the expression of miR biogenesis factors. Differential expression of miRs and miR biogenesis factors were identified.

## Results

### Tumor array

**Fig. 1:**
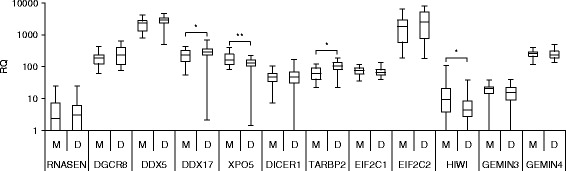
miR biogenesis factor expression by gene expression array in enucleated tumors with (*M*), *n* = 33, and without (*D*), *n* = 22, monosomy-3. The *box* represents the 25th and 75th percentiles, the *horizontal lines* represent the median, and the *whiskers* represent the minimum and maximum. *Brackets with an asterisk* above indicate statistical significance *P* <0.05 , **P < 0.01, Wilcoxon rank-sum test

miR and gene expression profiles of 33 enucleated uveal tumors with monosomy-3 and 22 without were obtained. This analysis identified 26 miRs as discriminators; 19 were down-regulated >2.0-fold and six were upregulated >2.0-fold (Table 1). In this data set, 13 patients, all with tumor monosomy-3, had manifested metastatic disease clinically on follow-up. Eight of the 26 miRs identified were also differentially expressed in these patients (Table 1). The strongest associations with monosomy-3 were observed for under-expression of X-linked miRs. Neither X-linked miRs nor other any miRs were differentially expressed by tumors from the 31 males compared to the 24 females studied. Twenty-seven of the tumors evaluated were considered to have TILs, a potential source of miRs; 28 were not. None of the miRs differentially expressed in tumors with and without monosomy-3 was differentially expressed in tumors with and without TILs. Gene expression of 12 miR biogenesis factors were also profiled by microarray in the 55 enucleated uveal melanoma tumors. Tumors manifesting monosomy-3 were characterized by higher levels of *TARB2* and *DDX17* and lower levels of *XPO5* and *HIWI* (Fig. 1).

**Table 1:** Tumor miRs differentially expressed

miR	Chr	Monosomy-3 vs. disomy-3^a^ fold difference	Monosomy-3 vs. disomy-3^a^ *P*	Metastatic vs. nonmetastatic^b^ *P*	Plasma levels^c^	
					Monosomy-3	Disomy-3
Over-expressed in tumors with monosomy-3						
hsa-miR-135a*	3	80	0.0003	NS	ND	ND
hsa-miR-624	14	7.6	0.0003	0.0004	ND	ND
hsa-miR-449b	5	5.9	0.0005	NS	ND	ND
hsa-miR-142-5p	17	7.5	0.0006	NS	1236	1059
hsa-miR-92b	1	2.9	0.0009	NS	688	ND
hsa-miR-628-5p	15	4.8	0.001	NS	ND	ND
Under-expressed in tumors with monosomy-3						
hsa-miR-509-3-5p	X	615	0.00000008	0.0002	ND	ND
hsa-miR-508-3p	X	4672	0.0000003	0.0004	ND	ND
hsa-miR-514	X	2887	0.0000009	NS	ND	ND
hsa-miR-506	X	1308	0.000001	0.0008	ND	ND
hsa-miR-513a-5p	X	1674	0.000002	0.001	ND	ND
hsa-miR-507	X	61	0.000003	NS	ND	ND
hsa-miR-509-3p	X	818	0.000004	0.0008	ND	ND
hsa-miR-513b	X	75	0.00003	0.00002	ND	ND
hsa-miR-876-3p	9	81	0.00003	NS	ND	ND
hsa-miR-378*	5	5.1	0.0002	NS	ND	ND
hsa-miR-935	19	4.1	0.0003	0.001	ND	ND
hsa-miR-181a	9	7.4	0.0004	NS	546	ND
hsa-miR-99a	21	5.6	0.0009	NS	ND	ND
hsa-miR-194	1	4.5	0.001	NS	ND	ND
hsa-miR-592	7	4.1	0.001	NS	ND	ND
hsa-miR-1296	10	15	0.001	NS	ND	ND
hsa-miR-624*	14	7.5	0.002	NS	ND	ND
hsa-miR-140-5p	16	10	0.002	NS	ND	ND
hsa-miR-651	X	6.1	0.002	NS	ND	ND

*NS* not significant, *ND* not detectable

^a^Monosomy-3, *n* = 33; disomy-3, *n* = 22

^b^Metastatic, *n* = 13; nonmetastatic, *n* = 42

^c^Average signal intensity

### Plasma array

Plasma miR profiles of pooled samples from 10 patients with monosomy-3 and 10 without were analyzed using quantitative nuclease protection assay (qNPA). Of the 674 human miRs assayed, 96 were detectable in plasma. Compared to patients without, 11 miRs were elevated >2.0-fold and four were reduced >2.0-fold in patients with tumor monosomy-3 (Table 2). None of the miRs that was discriminatory in the tumor array met the level of discrimination set for the plasma array. Only two of the miRs over-expressed in the tumor array were quantifiable in plasma, miR-92b and miR-142-5p (Table 1). The 1.6-fold increase in plasma miR-92b was statistically significant (*P* <0.02); the 1.2-fold increase in plasma miR-142-5p was not (*P* <0.5). The only other miR measurable in blood was miR-181a, levels of which were increased in plasma while being under-expressed in tumors in the presence of tumor monosomy-3.

**Table 2:** Plasma miRs differentially expressed

miR	Chr	Monosomy-3^a^	Disomy-3^b^	*P*
Increased in patients with tumor monosomy-3				
hsa-miR-191	3	7456	1760	0.0000001
hsa-miR-93	7	3344	836	0.000001
hsa-miR-221	X	10411	3449	0.00006
hsa-miR-342-3p	14	962	ND	0.00007
hsa-miR-19b	13	2385	1017	0.0002
hsa-miR-199a-5p	19	1977	ND	0.0003
hsa-miR-25	7	1490	ND	0.0009
hsa-miR-27a	19	5182	1993	0.0009
hsa-miR-23a	19	4566	1886	0.001
hsa-miR-15b	3	1195	530	0.001
hsa-miR-223	X	10286	3413	0.002
Decreased in patients with tumor monosomy-3				
hsa-miR-1227	19	1686	10791	0.0000008
hsa-miR-663	20	2196	16206	0.00001
hsa-miR-654-5p	14	420	1148	0.00008
hsa-miR-1238	19	1561	6172	0.0001

^a^Average signal intensity, *n* = 10

^b^Average signal intensity, *n* = 10

*ND* not detectable

### Plasma miR quantification

**Fig. 2:**
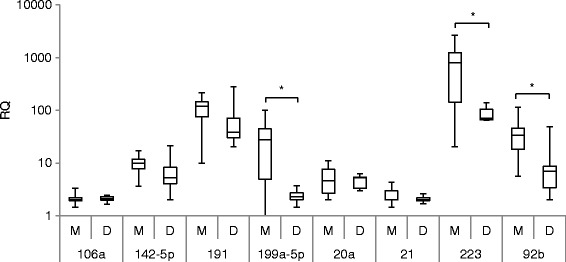
Plasma miR quantification by qRT-PCR in patients with enucleated tumors with (*M*), *n* = 10, and without (*D*), *n* = 10, tumor monosomy-3. The *box* represents the 25th and 75th percentiles, the *horizontal lines* represent the median, and the *whiskers* represent the minimum and maximum*. Brackets with an asterisk* above indicate statistical significance *P* < 0.05, Wilcoxon rank-sum test

**Fig. 3:**
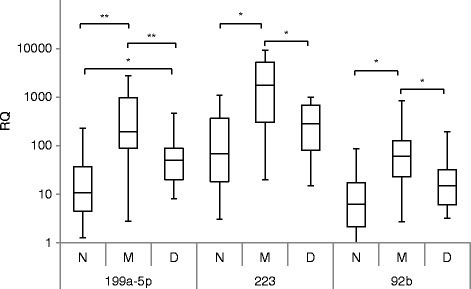
Plasma miR quantification by qRT-PCR in patients with (*M*), *n* = 33, and without (*D*), *n* = 32, monosomy-3 in which tumor chromosome 3 status was obtained on FNA biopsies. Also displayed are plasma levels of normal controls (*N*), *n* = 26. The *box* represents the 25th and 75th percentiles, the *horizontal lines* represent the median, and the *whiskers* represent the minimum and maximum. *Brackets with an asterisk* above indicate statistical significance *P* < 0.05, **P < 0.01, Kruskal-Wallis test

Plasma levels of select miRs increased in the tumor and in the pooled plasma arrays in the presence of tumor monosomy-3 were then examined by quantitative real-time polymerase chain reaction (qRT-PCR) in the individual patients tested, again 10 with tumor monosomy-3 and 10 without. The focus was on the two miRs that were over-expressed in the tumor array that were measurable in plasma, miR-92b and miR-142-5p, and three miRs elevated in the plasma array, miR-191, miR-199a-5p, and miR-223. Three miRs previously reported to be upregulated in uveal melanoma tumors compared to normal choroid, miR-20a, miR-21, and miR-106a, that were not differentially expressed in either the tumor or plasma array, were also assessed [5]. Differential expression in plasma as assessed by qRT-PCR paralleled the qNPA results (Fig. 2). miR-92b, miR-199a-5p, and miR-223 were significant higher in both the qNPA and the qRT-PCR analysis. miR-191 tended to be higher in the qRT-PCR analysis, but increases did not reach the level of significance (*P* < 0.10), as it did in the qNPA analysis. miR-142-5, miR-20a, miR-21, and miR-106a were not differentially expressed. Levels of the three miRs that were significantly different were then examined in another set of patients with primary uveal melanoma in which tumor chromosome 3 status was obtained on fine needle aspiration (FNA) biopsies. Levels of these miRs were also compared to those of 26 healthy donor controls. Plasma levels of miR-92b, 199a-5p, and 223 were significantly higher in patients with monosomy-3 when compared to patients with disomy; levels of all three were also higher when compared to levels of normal controls (Fig. 3).

## Discussion

Tumor and plasma miR profiling of patients with primary uveal melanoma was applied to investigate the role of epigenetic mechanisms in the metastatic process with an overall goal to develop blood biomarkers that could potentially help guide adjuvant therapy decisions and follow-up. Of 858 miRs assessed in tumors manifesting monosomy-3, an accurate predictor of the development of metastasis, 6 were found to be over-expressed and 20, under-expressed. The over-expressed miRs associated with monosomy-3 were analyzed by DIANA mirPath (Multiple microRNA Analysis), a web-based miR pathway analysis application [12]. The top three pathways potentially regulated were actin cytoskeleton, adherens junctions, and TGF-beta signaling, pathways implicated in metastasis, including in uveal melanoma [13–15]. Of note, whereas the target genes of most miRs are enriched, for example, on chromosomes 6, 16, 17, 19, and 22, miR target genes are not enriched on chromosome 3 [16]. None of the miRs we found to be differentially expressed in tumors with monosomy-3 was differentially expressed in tumors studied by Worley et al. [8], who used the class-2 GEP as a surrogate for metastasis. The most significant discriminators in our study were under-expression of miRs of the 506-514 cluster, which has been implicated in initiating melanocyte transformation and promoting melanoma growth and invasiveness [17, 18].

miR production is a complicated process requiring a large number of molecular events to be coordinated. None of the major miR biogenesis factors are encoded on chromosome 3. Tumors manifesting monosomy-3 were characterized by alterations in miR processing factors, which have been associated with the development of metastasis in several types of cancer. *DDX17* and *TARBP2* were upregulated, and *XPO5* and *HIWI* were down-regulated. *DDX17* (22q13.1), a nuclear endonuclease that produces 60 to 70 nucleotide pre-miRs, was identified as a metastasis-associated gene in renal cell carcinoma [19]. A decrease in exportin 5 (*XPO5*; 6p21.1), which transports pre-miRs into the cytoplasm, has been associated with prognosis in head and neck and in lung cancers [20, 21]. Upregulation of *TARBP2* (12q12-q13), a cytoplasmic endonuclease which cleaves pre-miRs into 21 to 22 nucleotide mature miRs in conjunction with Dicer (*DICER1*), has been associated with metastasis in breast cancer [22]. Down regulation of HIWI (12q24.33), which is integrated into the silencing complex, has been associated with metastasis in pancreatic cancer [23]. Alterations in Dicer, Drosha, and Gemin4, which have been observed in cutaneous melanoma, were not observed [24]. Most of the miRs identified that were discriminatory in tumors with monosomy-3 were down-regulated. How the alterations in miR biogenesis factors influenced this observation will require further study.

None of the miRs that we found to be discriminatory in the tumor array was found to be discriminatory in the plasma array. The plasma miRs most significantly increased was miR-191, which has been implicated in several oncogenic processes [25]. We were able to confirm using qRT-PCR that specific miRs differentially expressed in the arrays were increased in the plasma of patients with tumor monosomy-3 and significantly increased when compared to levels in normal donors. These included one miR over-expressed in the tumor array, miR-92b, and two increased in the plasma array, miR-199a-5p and miR-223. These miRs have also been implicated in several cellular processes. All three have been implicated in regulating genes that promote metastasis [26–28]. All three also regulate host responses. Of note, miR-92b belongs to a cluster of miRs that regulate T cells, including regulatory T cells [29], miR-199a-5p promotes regulatory T cells [30], and miR-223 regulates myeloid suppressor cells [31]. Regulatory T cells and myeloid suppressor cells have been implicated in uveal melanoma progression [32, 33]. Plasma levels of miRs reported to be upregulated in uveal melanoma tumors when compared to normal choroid, miR-20a, miR-21, and miR-106a, were also measured to examine the possibility that increases may represent circulating uveal melanoma cells, a potential predictor of metastasis [5, 34]. Levels of these miRs were not increased in the plasma of patients with tumor monosomy-3.

Virtually, all of the miRs discriminatory in the tumor array were not quantifiable in the plasma array. In contrast to tumor where more miRs were differentially under-expressed, more miRs were differentially increased in the plasma in patients with tumor monosomy-3 compared to without. That miR-expression patterns of tumor differ from those of blood has been previously reported [35]. Several mechanisms may generate blood miRs, including passive leakage from apoptotic or necrotic cells and active secretion of miR-containing microparticles and exosomes. These can occur in malignant cells but also in nonmalignant cells with a short half-life, such as blood cells, or upon tissue damage. There is evidence that most circulating miRs are blood-cell derived [36]. At least 100 different miRs have been shown to circulate in the blood of healthy donors, including most of the miRs we found to be differentially increased, miR-19b, miR-191, miR-199a-5p, miR-25, miR-23a, miR-223, and miR-93 [37, 38]. Several of the differentially expressed miRs identified have been previously reported to be elevated in the plasma or serum of patients with cancer, including miR-92b in prostate [39]; miR-223 in lung, esophageal, and hepatocellular [40–42]; and miRs-199a-5p, miRs-19b, miRs-15b, and miRs-25 in lung [40, 43, 44].

In addition to chromosome 3, abnormalities in chromosomes 1, 6, and 8 have also been associated with metastasis in uveal melanoma. Only three of the 26 tumor miRs and two of the 18 plasma miRs differentially expressed are located on these chromosomes. One of the miRs over-expressed in the tumor array, miR-135a*, localizes to chromosome 3. miR levels are regulated by several transcriptional and post transcription mechanisms as well as poorly understood degradation pathways [45]. miR-135a levels are regulated by Wnt/beta-catenin signaling [46]. Wnt/beta-catenin signaling has been implicated in uveal melanoma development [47]. The miR-506-514 cluster maps to the human X chromosome, which contains approximately 10 % of all miRs detected in the human genome. miR-223 and miR-221, which were increased in plasma, are also X-linked. Although the role of most has not yet been described, several X-linked miRs have been shown to have important functions in cancer as well as in immunity [48]. Nonrandom abnormalities have been previously observed on the sex chromosomes in uveal melanoma, but a consensus regarding their prognostic significance has not been established [49–51]. Although males manifest a slightly higher incidence and mortality rate, gender is not considered to play a major role in uveal melanoma predisposition or prognosis [52].

## Conclusions

These results, which derive from the largest number of uveal melanoma samples reported to date, support a role for epigenetic events mediated by miRs in uveal melanoma metastasis and further analysis of miRs as biomarkers of metastatic risk. They also suggest that potentially useful blood miRs may be derived from the host response as well as the tumor. Tumor monosomy-3 and class-2 GEP, although accurate predictors, are surrogate endpoints of metastatic death. Larger scale, prospective studies with clinical endpoints, including early compared to late metastases, will be necessary. The use of blood miR levels in conjunction with imaging studies as part of systemic surveillance for metastasis also merits study.

## Methods

### Patients

Tumors that had been cryopreserved from patients with uveal melanoma treated by enucleation at the Cleveland Clinic Cole Eye Institute between 2004 and 2010 were analyzed. Starting in 2009, blood was also collected from patients treated with enucleation and from patients undergoing FNA biopsy at the time of plaque radiotherapy. Computed tomography scans of the chest, abdomen, and pelvis were initially performed to rule out metastatic disease. Chromosome 3 status in the enucleation specimen was assessed by single nucleotide polymorphism array and in the FNA biopsies by fluorescent in situ hybridization as previously described [53]. Standard clinical and histologic characteristics were also assessed. This included the presence or absence of significant TILs, which was defined as being more than 100 lymphocytes in 20 high power (40×) fields [54]. All patients underwent scheduled surveillance for the development of metastases with clinical evaluation with liver imaging.

### Tumor miR array

Total RNA was extracted from 25 mg of cryopreserved tumor tissue using Trizol Reagent (Invitrogen, Carlsbad, CA) according to the manufacturer’s instructions and further purified using the miRNeasy Mini Kit (Qiagen, Valencia, CA). RNA quality was assessed using the Agilent 2100 Bioanalyzer, and concentration was measured using Nanodrop 1000 (Thermo Fisher Scientific, Waltham, MA). The total RNA (400 ng) was hybridized to the Illumina MicroRNA Profiling BeadChip, containing 858 mature human miR probes and 287 hypothetical small RNA probes according to the standard protocol.

### Tumor miR biogenesis factors

RNA was isolated from snap-frozen primary uveal melanoma tissue isolated from enucleated eyes. Purity and concentration were determined using a NanoDrop ND-1000 Spectrometer. Quality RNA was subsequently hybridized using a direct hybridization array kit (Illumina). Each RNA sample was hybridized using the HumanHT-12 BeadChip array (Illumina) in a multiple-step procedure; the chips were washed, dried, and scanned on the BeadArray Reader (Illumina). Raw microarray data were generated using BeadStudio v3.0 (Illumina). Microarray data analysis and quality control were performed using BeadArray R package v1.0.0. After background subtraction (using median background method), the data were normalized using quantile normalization and log-transformed.

### Plasma miR array

Plasma samples were forwarded to the HTG Molecular Diagnostics, Inc. (Tucson, AZ) for miR profiling using qNPA. The expression of 674 human miRs was analyzed using Whole Transcript miRNA Microarray Version 11. Each sample was tested in duplicate.

### Plasma miR quantification

The total RNA was isolated from plasma using the miRNeasy Mini Kit (Qiagen, Valencia, CA) according to the manufacturer’s instructions. With the exceptions of miR-142-5p and miR-92b, reverse transcription reactions were performed using a TaqMan MicroRNA Reverse Transcription Kit (Applied Biosystems, Foster City, CA) according to the manufacturer’s instructions. qRT-PCR was performed using the reverse transcription reaction product, TaqMan MicroRNA Assay kit, and TaqMan Universal PCR Master Mix (Applied Biosystems) according to the manufacturer’s instructions. TaqMan MicroRNA Assay kits for human miRs were used. Reactions were loaded onto a 96-well plate and run in duplicate on an ABI 7500 Fast Real-Time PCR System (Applied Biosystems). The reactions were incubated at 50 °C for 20 s and 95 °C for 10 min, followed by 40 cycles of denaturation at 95 °C for 15 s, then 1 min of annealing/extension at 60 °C. The ΔΔC_T_ method was used to determine relative number of copies (RQ) of miR. Data were normalized to a *Caenorhabditis elegans* synthetic miR sequence, cel-miR-39 (Qiagen), which was spiked in as a control during RNA isolation. The miScript PCR System from Qiagen (Valencia, CA) was used for quantification of miR-142-5p and miR-92b. miRs were isolated as described previously; 5 μL of isolated template RNA were used for subsequent reverse transcription reactions which were performed using the miScript II RT Kit according to the manufacturer’s instructions. Real-time PCR was performed using 2× QuantiTect SYBR Green PCR Master Mix, 10× miScript Universal Primer, 10× miScript Primer Assay, and template cDNA from reverse transcription; all reaction volumes suggested by the manufacturer were doubled to perform reactions in duplicate.

### Statistical analysis

Significance analysis of microarrays (http://statweb.stanford.edu/~tibs/SAM/) was used to identify miRs differentially expressed between monosomy- and disomy-3 tumors. Normalization by median centering and *t* test statistic were used in the analysis. In order not to miss subtly expressed miRs in tumors that may be measureable in plasma, the false discovery rate threshold for the tumor array was set at <0.01. Expression of tumor miR biogenesis factors was evaluated by Wilcoxon rank-sum tests. The miR array of plasma from patients with monosomy- and disomy-3 was analyzed by HTG Molecular Diagnostics, Inc. Data were normalized to the total signal for each microarray. Results are reported as average signal intensities. miRs were considered quantifiable if the average signal intensity was >526 in plasma from monosomy-3 donors and >531 in plasma from disomy-3 donors. Differentially expressed plasma miRs were selected using random variance *t* test, *P* <0.05, and absolute fold change >2.0. Differential expression of plasma RQ of specific miRs was assessed by Wilcoxon rank-sum test for comparison between two groups or Kruskal-Wallis test for comparison between multiple groups. All tests were two-sided with *P* < 0.05 considered significant.

## Abbreviations

FNA, fine needle aspiration; GEP, gene expression profiling; miR, microRNA; qNPA, quantitative nuclease protection assay; qRT-PCR, quantitative real-time polymerase chain reaction; RQ, relative number of copies
